# Secondary metabolite production and the safety of industrially important members of the *Bacillus subtilis* group

**DOI:** 10.1093/femsre/fuy028

**Published:** 2018-07-19

**Authors:** Colin R Harwood, Jean-Marie Mouillon, Susanne Pohl, José Arnau

**Affiliations:** 1 Centre for Bacterial Cell Biology, Institute for Cell and Molecular Biology, Newcastle University, Newcastle upon Tyne NE2 4AX, UK; 2 Department of Fungal Strain Technology and Strain Approval Support, Novozymes A/S, Krogshoevej 36, DK-2880 Bagsvaerd, Denmark

**Keywords:** polyketides, lipopeptides, nonribosomal peptides, *Bacillus*, toxicity

## Abstract

Members of the ‘*Bacillus subtilis* group’ include some of the most commercially important bacteria, used for the production of a wide range of industrial enzymes and fine biochemicals. Increasingly, group members have been developed for use as animal feed enhancers and antifungal biocontrol agents. The group has long been recognised to produce a range of secondary metabolites and, despite their long history of safe usage, this has resulted in an increased focus on their safety. Traditional methods used to detect the production of secondary metabolites and other potentially harmful compounds have relied on phenotypic tests. Such approaches are time consuming and, in some cases, lack specificity. Nowadays, accessibility to genome data and associated bioinformatical tools provides a powerful means for identifying gene clusters associated with the synthesis of secondary metabolites. This review focuses primarily on well-characterised strains of *B. subtilis* and *B. licheniformis* and their synthesis of non-ribosomally synthesised peptides and polyketides. Where known, the activities and toxicities of their secondary metabolites are discussed, together with the limitations of assays currently used to assess their toxicity. Finally, the regulatory framework under which such strains are authorised for use in the production of food and feed enzymes is also reviewed.

## Teaser

A review of the nomenclature, synthesis and structure of secondary metabolites produced by commercially important strains of bacteria, and the safety regime under which they operate.

## INTRODUCTION

*Bacillus licheniformis* and *B. subtilis* are two of the most commercially important bacteria, used for the production of a range of metabolites (vitamins, amino acids and antibiotics) and industrial enzymes (Harwood 1992). They are closely related to other members of the *B. subtilis* species complex (*B. subtilis* group) that also includes *B. amyloliquefaciens, B. atrophaeus, B. mojavensis, B. paralicheniformis, B. pumilus, B. tequilensis B. vallismortis* and *B. velezensis.* They are Gram-positive spore-forming members of the phylum Firmicutes and, in recent years, their phylogeny has undergone sweeping changes. As a result, there are frequent misnaming and name changes in the literature (Zeigler and Perkins 2015). Members of the group are widely distributed in soil where they help to recycle carbon and nitrogen via the production and secretion of macromolecular hydrolases such as proteases, amylases and cellulases.

Members of the *B. subtilis* group have long been known to produce a range of secondary metabolites, including polyketides (PKs), terpenes and siderophores, as well as ribosomally and non-ribosomally synthesised peptides. For decades the identification of secondary metabolites and antimicrobial peptides (AMPs) was based primarily on their extraction from the culture medium, often because of their inhibitory effect on other bacteria and fungi. This was followed by analysis of their chemical composition and structure, and subsequent identification of the genes involved in their synthesis. In the post-genomics era, reverse genetics approaches tend to be applied. This involves the use of bioinformatical tools to identify genes/gene clusters with similarities to genes involved in secondary metabolite synthesis already identified and characterised in the literature or in protein databases. In this review, we have used a web-based tool (antiSMASH 3.0, see later) to detect such clusters in members of this group that are of commercial interest and for which complete genomes are available at the National Center for Biotechnology Information (NCBI). However, it is important to emphasise that the presence of a gene cluster in a particular strain does not necessarily mean that the strain in question is capable to producing the expected secondary metabolite. There are many reasons for this, including gene silencing and a lack of knowledge about the environmental conditions needed to activate the cluster.

Analysis of the literature associated with secondary metabolites reveals a significant number of ambiguities, with identical sets of genes being identified as responsible for the synthesis of differently named metabolites. This problem results primarily from three issues; (i) the extraordinarily large range of structures and structural modification these compounds are subjected to in nature, (ii) in the absence of detailed chemical analyses, and (iii) the use of DNA/protein homology search programmes such as Blast to ‘identify’ secondary metabolite genes/gene clusters and thereby to predicted their metabolic products. The latter is often referred to as the ‘genome annotation issue’ in which homology between genes and operons is wrongly interpreted as indicating identical functionalities (Klimke *et al.*^2011^).

In contrast to primary metabolites, secondary metabolites are small organic molecules that are normally non-essential for the growth and development of the producing organism, but which contribute to their fitness over an evolutionary time scale. It is likely that secondary metabolites have been produced for over 500 million years, dating back to the Cambrian period (Baltz 2008; Cox and Wright 2013). In many cases their synthesis results from evolutionary pressures associated with the production of antimicrobial secondary metabolites by competitor organisms in the same environment (Perry and Wright 2013).

Secondary metabolites have a wide range of functions and activities, and this review surveys the literature on the secondary metabolites produced by *B. licheniformis* and *B. subtilis.* Both are commercially important species, being used for the production of industrial enzymes, vitamins, amino acids and other products that are used in food, beverages and health products, as well as products used in large-scale processes such as brewing, biofuel production and starch processing. Because of the nature of secondary metabolites, the existing literature is highly dispersed and, in some cases, ambiguous. The aim of this review is therefore to bring together issues such as nomenclature, structure, activity and, where appropriate, toxicity, to provide a single source for this information.

*Bacillus licheniformis* and *B. subtilis* are widely distributed in soil where they help to recycle nutrients via the production and secretion of macromolecular hydrolases such as proteases, amylases, phosphatases and cellulases. Their generally accepted lack of pathogenicity (de Boer and Diderichsen 1991; Pedersen *et al.*^2002^), combined with extraordinary capacity to secrete proteins and enzymes into the culture medium, has resulted in their widespread commercial exploitation (Harwood 1992). The primary reservoir of members of the *B. subtilis* group is the soil and associated phylloplane and rhizosphere (Borriss *et al.*^2018^). However, the soil represents a challenging environment with respect to the discontinuous supply of nutrients, abiotic stresses (osmolarity, water relations, pH, radiation, etc.) and competition from cohabiting microbes. To this end, members of the *B. subtilis* group have developed a range of strategies aimed at increasing their competitiveness and survival (Hecker and Völker 2004; Voigt *et al.*^2014^). These include so-called bet-hedging strategies in which the cell population differentiates into a variety of morphological and physiological cell types, each with specific functional roles in aiding the survival of the population as a whole rather than that of individual cells (Veening, Smits and Kuipers 2008; Grimbergen *et al.*^2015^). These differentiated cell types include spores, competent cells, biofilm-forming cells and motile cells, as well as cells that produce antimicrobial metabolites and peptides, cannibalistic toxins and macromolecular hydrolases. Spore formation is usually regarded as a ‘last resort’ response to nutrient deprivation and stress (Tocheva, Ortega and Jensen 2016).

It is worth noting that soil-dwelling organisms that generate spores (e.g. actinomycetes bacilli and fungi) are among the most prolific producers of antimicrobial compounds, in part reflecting aspects of the sporulation process itself. Sporulation is generally induced in response to nutrient deprivation and stress, and the extensive morphological differentiation processes that accompany sporulation necessarily consume both energy and nutrient resources (Tocheva, Ortega and Jensen 2016). In a population undergoing sporulation, a significant portion of the population is literally sacrificed to provide the nutrients and energy resources for the very much smaller proportion of sporulating cells and, as a result, the induction of sporulation is population density dependent (González-Pastor 2011). In the case of *Bacillus*, maximally ∼10% of the cells form spores and a larger portion of the non-sporulating cells in the population are lysed to provide the necessary nutrients; similar processes occur in filamentous bacteria and fungi where the substrate mycelium is sacrificed to provide these nutrients for the aerial mycelia on which the spores develop. However, this sudden release of nutrients is not only available to the organism itself but potentially also to competitor organisms cohabiting the same environment. To reduce this competition, sporulating microbes almost invariably synthesise antimicrobial metabolites aimed at restricting the growth of competitors during this vulnerable stage in their life cycle. These metabolites include peptides and PKs, as well as antibiotic compounds such as β-lactams and aminoglycosides that are widely used for the treatment of infections.

In addition to protecting the organisms during the vulnerable stages of sporulation, and increasing their competitiveness, *Bacillus* species also synthesise a variety of other bioactive secondary metabolites that, for example, facilitate colonisation (e.g. attachment, swarming, etc.) and help recover trace elements from the environment (e.g. siderophores). Still other species have toxins, such as the non-ribosomally synthesised cyclic dodecadepsipeptide ionophore, cereulide, responsible for *B. cereus*-mediated gastrointestinal disease (Agata *et al.*^1995^). In recent years, the roles of secondary metabolites in biological control, and the use of strains producing such metabolites as probiotics, have become an area of considerable research activity as they provide low technological and environmentally sustainable approaches for plant growth promotion, the inhibition of pathogens and improvements in the nutritional value of animal feeds (Gao *et al.*^2015^; Hinarejos *et al.*^2016^).

This review focuses primarily on two classes of secondary metabolites synthesised by members of the *B. subtilis* group: PKs, synthesised by polyketide synthases (PKS), and peptides, synthesised by non-ribosomal peptide synthetases (NRPS). The reaction intermediates of both pathways are characterised by their retention, via thioester linkages, within their respective macromolecular assembly machines. As a result, they are referred to generically as ‘thiotemplate modular systems’ (TMS). Because they exhibit some structural and functional similarities to non-ribosomally synthesised peptides and PKs, some ribosomally synthesised AMPs that are commonly produced by members of this group are also briefly discussed. Because strains encoding these metabolites are used for the production of food and feed enzymes, the regulatory framework under which such strains are authorised is also reviewed.

## POLYKETIDES SYNTHASES AND NON-RIBOSOMAL PEPTIDE SYNTHETASES

PKS and NRPS are molecular assembly machines that use macromolecular protein complexes, rather than nucleic acid templates, to direct the synthesis of their target products. The assembly is initiated through the activity of phosphopantetheinyl transferases (PPTases). PPTases convert the inactive *apo* forms of the modular enzymes involved to the active *holo* forms of, in the case of both PKS and fatty acid synthase assembly lines, their cognate acyl carrier proteins (ACP) and, in the case of NRPS assembly lines, their cognate peptidyl carrier proteins (PCP) (Donadio, Monciardini and Sosio 2007). Subsequent assembly involves a series of enzymatic reactions, the intermediates of which remain covalently attached to the complex as thioesters to a phosphopantetheine prosthetic group. Separate modular domains within the complex add each substrate monomer in turn and, as a result, there are at least as many modules as there are monomers incorporated in the final product. In addition, dedicated ‘tailoring enzymes’ are often encoded within the biosynthetic gene cluster. These enzymes function to provide alternative amino acid building blocks, to carry out modifications to elongating chains while still attached to the assembly machine, or to carry out post-assembly modifications. The modular nature of PKS and NRPS assembly pathways, and the presence of tailoring enzymes, means that they are able to synthesise an extremely wide diversity of secondary metabolites and structural characterisation requires detailed chemical analysis (Caboche *et al.*^2010^; Wang *et al.*^2014^; Weissman 2014).

Prior to the genomics’ era, strains encoding these two classes of compound were identified primarily via their biological activity against other organisms. Nowadays strains with the potential to synthesise these compounds can be identified by the presence in their genomes of easily identifiable PKS and NRPS gene clusters that encode the large multimodular polypeptides required for their synthesis. Genome mining has become a key tool in the identification of such gene clusters, and the BAGEL3 (van Heel *et al.*^2013^; http://bagel.molgenrug.nl) and antiSMASH (Weber *et al.*^2015^; http://antismash.secondarymetabolites.org) web servers provide a comprehensive set of tools to automate this process. Associated with antiSMASH is a database that facilitates queries for specific biosynthetic gene cluster types (Blin *et al.*^2016^).

In the case *B. subtilis* 168, two valuable integrated databases have been developed, BSubCyc (Caspi *et al.*^2014^), part of the BioCyc database collection, and SubtiWiki (http://subtiwiki.uni-goettingen.de; Zhu and Stülke 2018). Access to BSubCy is now behind a pay wall, while SubtiWiki is freely available. SubtiWiki is a relational database providing genome and regulatory browsers, published information on genes and their products, interactive metabolic pathways and interactions networks. Despite being based on the model organism, *B. subtilis* strain 168, similarities within members of the *B. subtilis* group means that both databases are valuable resources for other strains within the group.

## SYNTHESIS OF NRP

*Bacillus* species produce a wide range of peptides that are synthesised by NRPS. These peptides have a variety of forms and functions, including iron-chelating siderophores, biosurfactants, cytotoxic cyclic lipopeptides and clinically important peptide antibiotics. In addition, several *Bacillus* strains with antibacterial and/or antifungal activities are used as biocontrol agents in agriculture, with three families of cyclic lipopeptides being of particular importance, namely surfactins, iturins and plipastatins/fengycins (Ongena and Jacques 2008).

**Figure 1.**
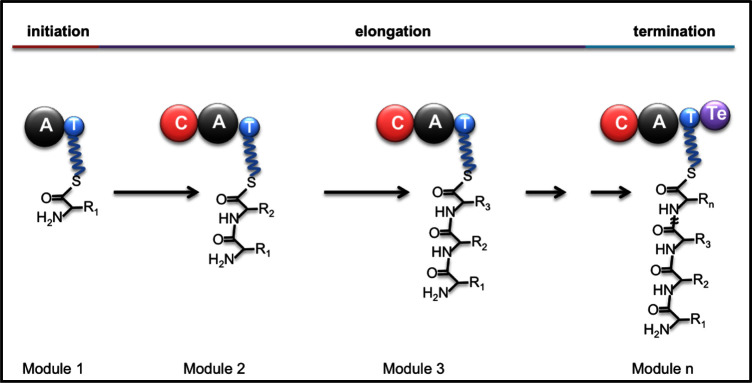
Non-ribosomal peptide (NRP) synthesis. The following domains are involved: A, adenylation; C, condensation; T, peptidyl carrier protein (PCP); Te, termination domain with thioesterase (TE) activity (modified from Donadio, Monciardini and Sosio 2007).

Amino and hydroxy acids are the basic building blocks for NRP, linked by amide or ester bonds, respectively. Each NRPS complex has a loading module, a variable number of elongation modules and a termination module (Fig. 1). The loading module has an adenylation (A) domain that selects the first amino or hydroxy acid building block, activates it as an amino acyl adenylate and transfers it to a PCP or T domain where it attaches via a thioester bond. In addition to A and T domains, the subsequent elongation modules additionally have a condensation (C) domain, responsible for peptide/ester bond formation between the amino/hydroxy acid present on its T domain and the peptidyl intermediate bound to the T domain of the preceding module. Finally, the termination (Tc) module has a thioesterase (TE) domain that releases the NRP from the complex (Donadio, Monciardini and Sosio 2007). The diversity of NRP structures is further expanded by the presence of additional modules that carry our specific modifications to the basic structural elements, such as amino acid epimerisation (E), methylation (M), reduction (R) and the replacement of C domains with heterocyclisation domains (Cy) (Donadio, Monciardini and Sosio 2007).

## SYNTHESIS OF PKs

PKs are a structurally diverse family of secondary metabolites that exhibit a wide range of biological activities. The PKs discovered so far number into the thousands. They are broadly classified into three structural classes according to the characteristics of the products of the gene clusters responsible for their synthesis (Hutchinson 1999):

- Type I PKS, consisting of one or more multifunctional proteins that contain a different active site for each enzyme-catalysed reaction in PK carbon chain assembly and modification;
- Type II PKS, aggregates of monofunctional proteins that are used iteratively in the biosynthesis of multi-aromatic products;
- Type III PKS, members of the chalcone synthase and stilbene synthase protein superfamilies. In contrast to type I and type II PKSs, they use free CoA thioesters as substrates and therefore do not require the involvement of 4^΄^-phosphopantetheine residues or ACP.

**Figure 2.**
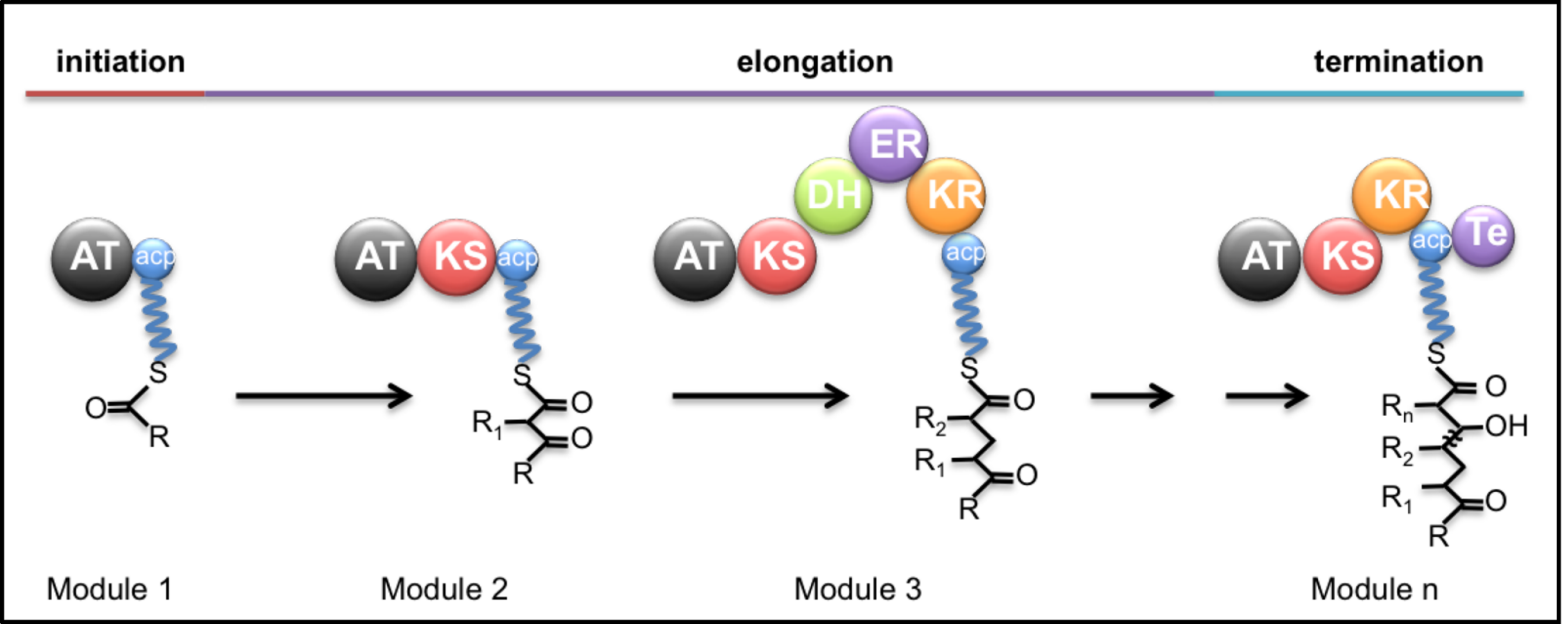
Polyketide (PK) synthesis. The following domains are involved: ACP, acyl carrier protein; AT, acyltransferase; DH, dehydratase; ER, enoylreductase; KR, β-ketoreductase; KS, ketosynthase; Te, termination domain with thioesterase (TE) activity (modified from Donadio, Monciardini and Sosio 2007).

PKS generate PKs via the oligomerisation of carboxylic acids. The multi-domain PKS consist of a series of modules that provide the template on which PK synthesis takes place. Synthesis is initiated at the loading module, continued by a variable number of elongation modules, and terminated at a release module (Fig. 2). Each elongation module consists of at least three domains: an acyltransferase (AT) domain, a ketosynthase (KS) domain and an ACP domain. The loading module lacks a functional KS domain, while the release module contains an additional TE domain that releases the completed PK from the complex. At each module in the complex, the AT domain selects the required chain extender unit (usually malonyl-CoA or methylmalonyl-CoA) and transfers it to the ACP domain where a thioester bond is formed. The KS domain then catalyses a decarboxylative condensation between the extender unit on the same module and the PK intermediate bound to the ACP domain of the preceding module. In addition to the core domains, individual PKS modules may also contain domains that catalyse specific extender unit modifications (e.g. β-ketoreductase [KR], dehydratase [DH], enoylreductase [ER], methylase [M]) (Donadio, Monciardini and Sosio 2007). These modifications contribute to the structural diversity and activity spectra of PKs.

## MEMBERS OF THE *B. SUBTILIS* GROUP ENCODE A NUMBER OF PKS AND NRPS GENE CLUSTERS

Sixty-eight strains of *B. subtilis* have been fully sequenced (NCBI Genomes Database December 2017), with genome sizes ranging from 3.88 to 4.30 Mb. The genome of the model Gram-positive bacterium *B. subtilis* strain 168 (4.21 Mb) encodes three NRPS gene clusters and one hybrid PKS/NRPS gene cluster (NC_0009643; Kunst *et al.*^1997^, Borriss *et al.*^2018^). The three NRPS gene clusters encode the catechol-based iron-chelating siderophore bacillibactin (*dhb* gene cluster), the lipodecapeptide plipastatin (*pps* gene cluster) and the lipoheptapeptide surfactin (*srf* gene cluster). The *pks* cluster encodes bacillaene, a hybrid linear PKS/NRP. However, strain 168 has an inactive form of the 4-phosphopantetheinyl transferase (PPTase) gene (pseudogenes *sfp/1, sfp/2*: Tsuge, Ano and Shoda 1996, 1999) required for the activation of TMS enzymes, and is therefore unable to produce surfactin, plipastatin or bacillaene. The production of these lipopeptides can be restored by transformation with a functional ectopic copy of the *sfp* gene (Ongena *et al.*^2007^).

Reports in the literature indicating that *B. subtilis* strain 168 produces the antifungal peptide fengycin have been revised in recent years with the identification of the product of the *pps* gene cluster as plipastatin, an NRP that is closely related to fengycin (Stein 2005). Similar issues appear throughout the literature as a result of automated annotation programs that replicate older, outdated or inaccurate annotations. The results of a bioinformatical analysis of the genomes of the 68 strains for the presence of NRPS and PKS gene clusters are summarised in Table 1 and given in detail in Table S1 (Supporting Information). NRPS gene clusters encoding surfactin, bacillibactin and plipastatin/fengycin synthesis are universally present (with the possible exception of strain KCTC 3135 that appears to lack a plipastatin/fengycin gene cluster). AntiSMASH reported *B. subtilis* strain Bs-115 (NZ_CP020722.1) as encoding two plipastatin/fengycin gene clusters. However, further analysis revealed that its genome sequence started in the middle of this strain's sole plipastatin/fengycin gene cluster. Seventy-seven percent of the strains have a gene cluster coding for the hybrid PKS/NRP, bacillaene.

**Table 1.** Summary of the secondary metabolites clusters (non-ribosomal peptides and polyketides) identified in complete genomes of *B. amyloliquefaciens, B. licheniformis, B. paralicheniformis, B. subtilis* and *B. velezensis*, identified by genome mining using antiSMASH (Weber *et al.*^2015^).

Species and type	Compound	Prevalence
*Bacillus subtilis* (n = 68)		
Non-ribosomal peptides		
	Surfactin	99%
	Plipastatin/Fengycin	97%
	Bacillibactin	99%
	Bacilysin	93%
	Locillomycin	2%
	Xenocoumacin	2%
	Pelgipeptin	2%
	Tridecaptin	2%
Polyketides		
	Bacillaene	77%
	Macrolactin	6%
	Difficidin	6%
	Kalimantacin/Batumin	2%
*Bacillus amyloliquefaciens* (n = 21)		
Non-ribosomal peptides		
	Surfactin	100%
	Plipastatin/ Fengycin	95%
	Bacillibactin	100%
	Bacilysin	100%
	Locillomycin	5%
	Iturin group	100%
Polyketides		
	Bacillaene	100.0%
	Macrolactin	71%
	Difficidin	67%
*Bacillus velezensis* (n = 47)		
Non-ribosomal peptides		
	Surfactin	100%
	Plipastatin/ Fengycin	100%
	Bacillibactin	100%
	Bacilysin	100%
	Locillomycin	11%
	Tridecaptin	2%
	Iturin group	100%
Polyketides		
	Bacillaene	100%
	Macrolactin	100%
	Difficidin	100%
	Kalimantacin/Batumin	6%
*Bacillus licheniformis* (n = 14)		
Non-ribosomal peptides		
	Lichenysin	100%
	Bacillibactin-like	100%^a^
	Aerobactin-like	100%
Polyketides		
	Chalcone-like^b^	93%
*Bacillus paralicheniformis* (n = 6)		
Non-ribosomal peptides		
	Lichenysin	100%
	Bacillibactin-like	100%
	Aerobactin-like	100%
	Plipastatin/Fengycin	100%
	Bacitracin	100%
Polyketides		
	Chalcone-like^b^	100%

The identified compound names reflect homology to well characterised gene clusters, but actual products could be different, for example due to the synthesis of modification proteins.

a One strain encoded two bacillibactin-like gene clusters.

b Encodes a chalcone and stilbene synthase domain protein.

Gene clusters for a number of other classes of AMPs/secondary metabolites were also identified. The most frequently found were the antimicrobial dipeptides, bacilysin and rhizocticin, which were encoded by 93% and 46% of the strains, respectively. Similarly, the ribosomally synthesised AMPs, subtilosin A and sublancin, are encoded by 93% and 22% of the strains, respectively. Strain *B. subtilis* subsp. *inaquosorum* DE111 (NZ_CP013984.1) exhibited an atypical secondary metabolite profile and therefore its taxonomy needs to be investigated further.

*Bacillus amyloliquefaciens* is closely related to and was often misidentified in the literature as *B. subtilis.* More recently, a similar confusion has arisen for its close relative, *B. velezensis*, leading to historical strain misidentifications in the literature (Dunlap *et al.*^2016^). It is therefore not possible to be sure whether or not sequences annotated as *B. amyloliquefaciens* in the NCBI database are indeed correctly identified. There is currently a great deal of interest in the commercial applications of *B. amyloliquefaciens* and *B. velezensis* strains as biocontrol agents and, as a result, we have used antiSMASH to analyse 21 fully sequenced genomes of *B. amyloliquefaciens* strains and 48 fully sequenced genomes of *B. velezensis* in the NCBI Genomes database (April 2018). The most common NRP and PKS biosynthetic gene clusters detected by antiSMASH were surfactin, plipastatin/fengycin, bacillibactin, iturin compounds, bacilysin and bacillaene (Table 1 and Table S1, Supporting Information). It is worth noting that the identification of iturin group clusters by antiSMASH proved to be problematical as the plipastatin/fengycin and iturin gene clusters were adjacent to each other on the chromosome and former was invariably ‘called’ in preference to the latter. Analysis of the individual ‘plipastatin/fengycin’ clusters within the antiSMASH programme was the only way to identify the presence of the iturins cluster.

Approximately 70% of *B. amyloliquefaciens* strains and 100% of the *B. velezensis* strains encode the PKs macrolactin and difficidin, both of which have antimicrobial activities. Macrolactins are 24-membered ring lactones modified by the attachment of groups such as glucose β-pyranoside (Schneider *et al.*^2007^). Difficidin is an unsaturated 22-membered macrocylic polyene lactone phosphate ester (Zimmerman *et al.*^1987^; Argüelles-Arias *et al*^2009^). All of the strains encoded a PKS-like gene cluster with a low level (7%) similarity to that encoding a butirosin-like thiopeptide (Llewellyn, Li and Spencer 2007). A few strains (5%–11%) of both species encode a gene cluster similar to the PK Kijanimicin, a spirotetronate antibiotic with a broad spectrum of antimicrobial activity against Gram-positive bacteria and some strains (6%) of *B. velezensis* encoded Kalimantacin/Batumin-related gene cluster which, like Bacillaene, is formed via a hybrid PKS-NRPS pathway.

As with *B. subtilis*, a number of other secondary metabolite clusters were identified in a small number of strains of *B. amyloliquefaciens* and *B. velezensis* (4%–14%), most commonly the lantibiotic mersacidin and the phosphono-oligopeptide rhizocticin. *Bacillus amyloliquefaciens* strain UMAF6639 (NZ_CP006058.1) did not exhibit the typical secondary metabolite profile associated with this species and a Blastn analysis showed only limited homology to other *Bacillus* species. This strain was therefore omitted pending confirmation of its identification.

The genomes of 14 strains of *B. licheniformis* (Rey *et al.*^2004^; Dunlap *et al.*^2015^) have been fully sequenced and annotated (NCBI Genomes Database July 2018). An additional strain, SRCM1101441 (NZ_CP021507.1), exhibited a secondary metabolite profile that matches that of *B. subtilis* and this was confirmed by Blastn analysis. The *B. licheniformis* strains analysed tended to exhibit fewer secondary metabolite gene clusters than those of the other species (Table 1 and Table S2, Supporting Information)**.** Each of the strains encoded a gene cluster for the NRP lichenysin and a gene cluster closely related (>50% identity) to the *B. subtilis dhb* cluster encoding bacillibactin. Unusually, strain SRCM100141 encodes two bacillibactin-like gene clusters, one very closely related to that in *B. subtilis*. Given the long association between bacitracin and *B. licheniformis*, a surprising observation was the absence of bacitracin gene clusters in these genomes, as noted previously for strain ATCC 14580 (Rey *et al.*^2004^). All but one of the strains encoded a chalcone/stilbene synthase domain protein, although there are no reports of strains of *B. licheniformis* synthesising this normally plant-specific PK (Tables 1 and Table S2, Supporting Information).

Gene clusters for a number of other classes of AMPs/secondary metabolites were also identified. All of the *B. licheniformis* strains encoded gene clusters for the lantipeptide lichenicidin VK21, an unidentified lassopeptide and aerobactin-like siderophore. The lichenicidin gene cluster of DSM13 (Veith *et al.*^2004^) actually includes two structural genes (*lanA1* and *lanA2*) as well as genes involved in its modification (*lanM, lanB, lanC, lanP*), regulation (*lanR, lanK*), export (*lanT(P*) and immunity (*lanE, lanF, lanG*). This gene cluster is therefore likely to produce a two-peptide antibiotic (Dischinger *et al.*^2009^). Two strains, BL-010 and HRBL-15TDI7, encode gene clusters for a subtilin-like lantibiotic.

The absence of bacitracin gene clusters in *B. licheniformis* led us to analyse the genomes of *B. paralicheniformis*, six fully sequenced and annotated strains of which have been deposited in the NCBI Genomes Database (July 2018). In addition to encoding NRPS gene clusters for lichenysin and bacillibactin- and aerobactin-like siderophores, each of the strain also encoded plipastatin/fengycin and bacitracin gene clusters. Like *B. licheniformis*, they too encoded a chalcone/stilbene synthase domain protein.

## CHARACTERISTICS OF THE MAIN PKS AND NRPS SYNTHESISED BY MEMBERS OF THE *B. SUBTILIS* GROUP

### Surfactin and lichenysin

**Figure 3.**
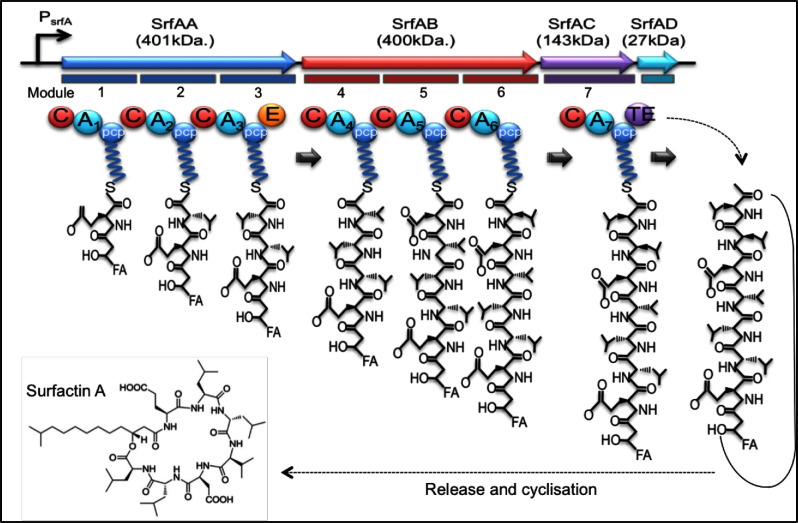
Surfactin synthesis. The multienzyme complex responsible for surfactin synthesis consists of seven modules, one each for the seven amino acids. These domains catalyse the 24 chemical reactions involved. The last domain is responsible for release and cyclisation surfactin (modified from Sieber and Marahiel 2005).

Although the synthesis and role of surfactin has been best studied in *B. subtilis*, related species also synthesise very similar NRPs (e.g. *B. amyloliquefaciens, B. licheniformis, B. mojavensis, B. pumilus*; Chen, Juang and Wei 2015). Surfactin is one of the most powerful known biosurfactants; at a concentration of 20 μM, surfactin decreases the surface tension of water from 72 to 27 mN/m. Surfactin consists of four isomers (surfactin A–D) that exhibit a wide variety of physiological activities. The chemical structure of surfactin includes a peptide loop of seven amino acids (L-asparagine/L-aspartate, L-leucine, L-glutamate, L-leucine, L-valine and two D-leucines), attached to a hydrophobic fatty acid chain, the length of which is isoform dependent (Fig. 3).

In *B. subtilis*, surfactin biosynthesis is regulated by a quorum-sensing system which crosslinks surfactin synthesis, competence and sporulation in a complex network of pheromones and pleiotropic regulators (Nakano, Xia and Zuber 1991). *Bacillus. subtilis* continuously secretes a prenyl-modified oligopeptide pheromone, ComX, which accumulates in the culture broth (Okada *et al.*^2015^). Upon reaching a critical cell density at around the onset of stationary phase, the membrane located sensor kinase ComP is activated, leading to the phosphorylation of its cognate response regulator, ComA (Nakano, Xia and Zuber 1991; Jacques 2011). Thereafter, activated ComA (ComA∼P) induces the transcription of the *srfA* operon comprising four open reading frames, namely *srfAA, srfAB, srfAC* and *srfAD.* However, as implied above, the transcription of the *srfA* operon is affected by a number of other regulators such as CodY DegU and AbrB, while the intracellular concentration of ComA∼P is strongly influenced by regulators belonging to the Rap and Phr family of phosphatases (Okada *et al.*^2015^).

Surfactin has non-specific cytolytic activity, although the composition of the target phospholipid bilayer influences its penetration (Deleu *et al.*^2003^). It lyses mammalian cells (including red blood cells) *in vitro* at concentrations of 40 μM– 60 μM; at concentrations up to 25 μM its cytolytic activity is not considered to be significant. The ability of surfactin to lyse cells is a feature of its surfactant activity and is a property that is shared with, for example, sodium lauryl sulphate (SLS), an anionic detergent widely used in domestic cleaning products. To put the ‘toxicity’ of surfactin into perspective, the feeding of oral doses of surfactin C to pregnant ICR mice at concentrations ranging from 0 to 500 mg/kg bw/day resulted in no maternal toxicity, fetotoxicity or teratogenicity (Hwang *et al.*^2008^). In contrast, toxicity studies of SLS in mice and rabbits, using an oral dose of 600 mg/kg bw/day, resulted in total resorption of foetuses, increased litter loss and/or abortion, together with severe maternal toxicity. At 300 mg/kg bw/day, no developmental toxicity was observed although slight-to-moderate maternal toxicity was observed (Blackburn *et al.*^2005^).

The ecotoxicity of surfactin was determined using the Microtox test, which measures the reduction in light emission of the marine bioluminescent bacterium *Vibrio fischeri*, and the *Daphnia magna* immobilisation test, which measures the immobility of this microcrustacean (Deravel *et al.*^2014^). In both cases, the ecotoxicity of surfactin was shown to be low, particularly compared with widely used fungicides.

Lichenysin is a non-ribosomally synthesised cyclic lipopeptide, similar in structure to surfactin. The gene cluster responsible for lichenysin is present in most strains of *B. licheniformis* (Madslien *et al.*^2013^). Several *in vitro* studies have indicated a strong correlation between the structure and properties of surfactin and lichenysin, including their activities in cytolytic and inhibitory assays (e.g. boar spermatozoa motility, Vero cells and haemolysis: Mikkola *et al.*^2000^; Nieminen *et al.*^2007^; Apetroaie-Constantin *et al.*^2009^). Lichenysin, inhibitory activity was generally observed at concentrations above 10 μg/ml for the boar spermatozoa assay and 33 μg/ml for the Vero cells assay. Haemoglobin release (≥50%) was only observed in samples containing >33 μg/ml (Madslien *et al.*^2013^).

The *in vitro* activities of surfactin and lichenysin are likely to be due to their non-specific detergent-like properties (Hoornstra *et al.*^2003^) and, in the absence of definitive animal studies, do not justify the use of the term cytotoxin to describe these compounds.

### Plipastatin and fengycin

The *pps* gene cluster of *B. subtilis* strain 168 has been described in the literature as being responsible for the synthesis of both fengycin and plipastatin (Honma *et al.*^2012^). Plipastatin and fengycin are biosurfactant antifungal cyclic lipodecapeptides that are closely related in structure, mode of synthesis and activity (Ongena *et al.*^2005^). They consist of a β-hydroxy fatty acid connected to the N-terminus of a decapeptide that includes four D-amino acids and the non-proteinogenic amino acid L-ornithine. The C-terminal residue (Ile) is linked to a tyrosine residue at position 3, forming the branching point of the acyl peptide and the eight-membered cyclic lactone. Although originally isolated independently in 1986, recent NMR studies carried out to resolve the structural nomenclature of these compounds found them to display only minor structural variations under different salt conditions (Honma *et al.*^2012^), For the sake of clarity, in this report the name plipastatin is used. The exact mode of action of plipastatin is not fully understood but seems to involve the inhibition of phospholipase A_2_ and the formation of pores in fungal membranes. The use of plipastatin-like compounds has been widely advocated as replacements for chemical fungicides because of their biodegradability and lack of reported toxicity to plants and animals.

**Figure 4.**
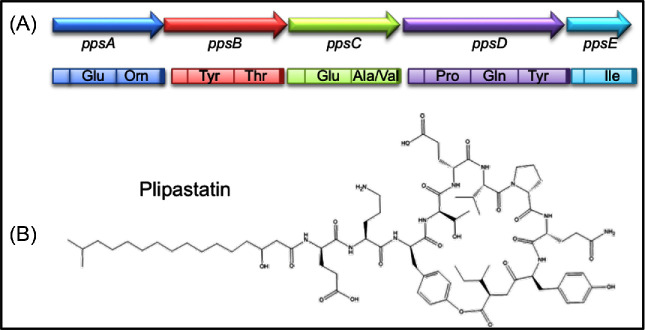
Plipastatin structure and synthesis. (**A**) The *ppsABCDE* operon of *B. subtilis* and structure of the core peptide. (**B**) The cyclic lipodecapeptide structure of the plipastatin.

Many strains of *B. subtilis* and *B. licheniformis* have NRPS gene clusters that encode plipastatin synthesis. In *B. subtilis* 168, the *ppsABCDE* gene cluster is 384 kb in length and encodes five peptide synthetases, namely PpsA (289 kDa), PpsB (290 kDa), PpsC (287 kDa), PpsD (407 kDa) and PpsE (145 kDa) (Fig. 4).

### Bacillaene

**Figure 5.**
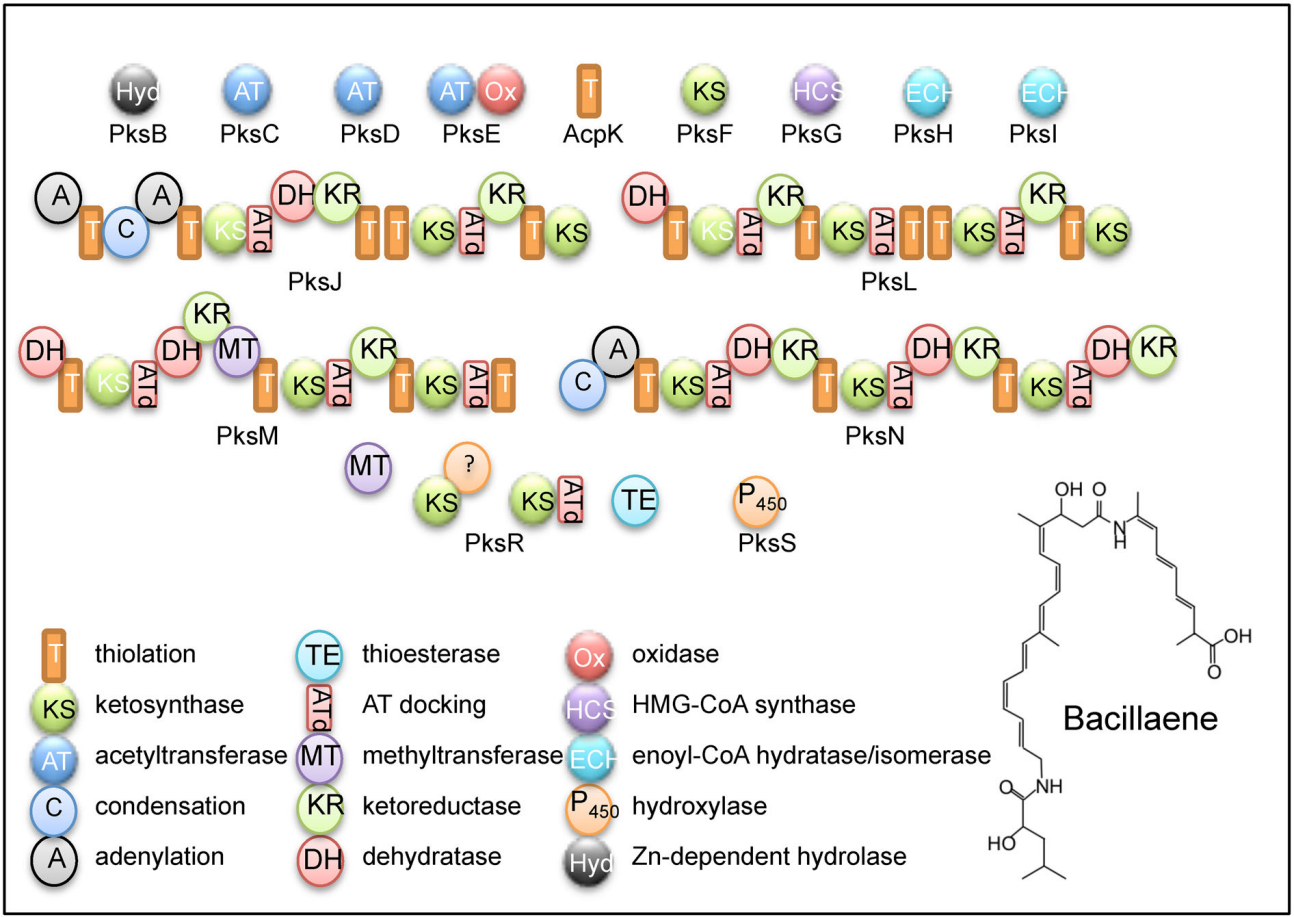
Bacillaene and the bacillaene gene cluster. PksB, PksC, PksD, PksE, PksF, PksG, PksH, PksI, AcpK and PksS are free-standing enzymes involved in *trans* to the multimodular proteins. PksJ, PksL, PksM, PksN and PksR are multimodular NRPS/PKS (PksJ, PksN) or PKS (PksL, PksM, PksN) proteins forming the core assembly line synthase (modified from Straight *et al.*^2007^).

Bacillaene is a bacteriostatic antibiotic, inhibiting rather than killing its target. It is active against a broad spectrum of bacteria, including cyanobacteria (Butcher *et al.*^2007^; Wu *et al.*^2014^). It does so by inhibiting prokaryotic, but not eukaryotic, protein synthesis (Patel *et al.*^1995^). Based on the analysis of an orthologous *bae* gene cluster of *B. amyloliquefaciens* FZB42 (Moldenhauer *et al*. 2007, 2010), the 16-gene 80-kb *pks* gene cluster, occupying 2% of the *B. subtilis* 168 genome, encodes the ∼25 megadalton hybrid NRPS/PKS complex that synthesises bacillaene (Fig. 5). Related clusters are present in 77% of sequenced strains of *B. subtilis* and all currently sequenced strains of *B. amyloliquefaciens* (Table 1). The bacillaene NRPS/PKS complex is large enough to be visualised by cryoelectron microscopy (Straight *et al.*^2007^). The structure of bacillaene has been solved and a model proposed for its biosynthesis (Straight *et al.*^2007^). The bacillaene synthase gene cluster reveals features in common with those from streptomycetes, myxobacteria and cyanobacteria, including three *trans*-acting AT domains that introduce substrates to the assembly line and a six-protein subcluster that converts a carbon–oxygen double bond to a β-methyl group (Moldenhauer *et al.*^2010^). There are no reports on the toxicity of bacillaene to higher organisms and this compound is included in a patent for possible use as an anti-acne agent (Eskandarian 2009).

### Iturin group

The iturin group is a large family of cyclic heptapeptides with a C14-C17 aliphatic β-amino fatty acid. They have chiral peptide sequences of L- and D- amino acids (LDDLLDL) and are cyclised by the formation of an amide bond between the N‐terminal β‐amino fatty acid and the C‐terminus of the peptide. The group includes iturin (variants A, C, D and E), bacillomycin (D, F, L and Lc) and mycosubtilin, as well as other variants with names that reflect their bacterial source (e.g. mojavensin). The β-amino fatty acid linked to the amino acid sequence Asn-Tyr-Asn is a common characteristic of the iturin group (Duitmann *et al.*^1999^; Moyne, Cleveland and Tuzun 2004).

**Figure 6.**
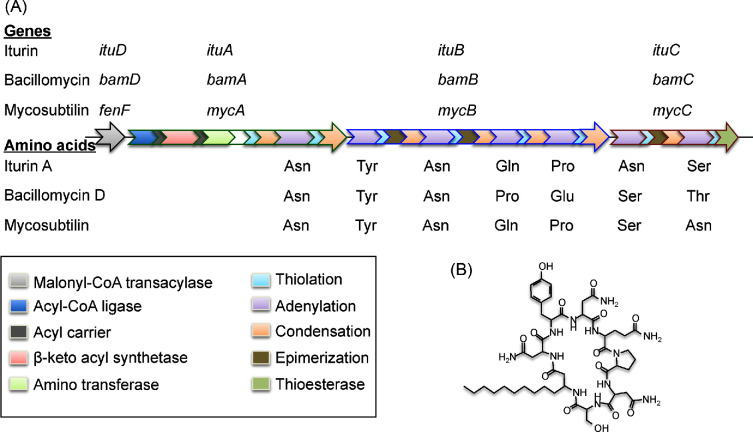
Iturin group gene cluster. (**A**) Representative 4-gene iturin operon showing the gene names and amino acid sequences for iturin, bacillomycin and mycosubtilin, colour coded to identify the activities of the various domains. (**B**) Structure of iturin (modified from Duitmann *et al.*^1999^).

Iturins are synthesised by an ∼38 kbp NRPS operon comprising four genes (Fig. 6; Duitmann *et al.*^1999^). The similarities between iturin group gene clusters are so close that we have not distinguished between them in Table 1 and Table S1 (Supporting Information). Iturin production is strongly associated with *B. amyloliquefaciens* and closely related species such as *B. velezensis*. All members of the iturins group have strong antifungal activity against a number of important fungal pathogens (e.g. *Rhizoctonia*, *Penicillium*, *Aspergillus*, *Fusarium* and *Pyricularia*) and, consequently, strains producing these compounds are being developed as potential biocontrol and plant growth promotion agents. For example, iturin A displays strong fungicidal activity against *Fusarium graminearum*, completely killing conidial spores at a minimal inhibitory concentration of 50 μg/ml (Fickers *et al.*^2009^; Gong *et al.*^2015^). The antifungal activity of iturins is related to its interaction with the cytoplasmic membrane of target cells, resulting in the formation of ion-conducting pores and increased K^+^ permeability. Both the lipid composition of the target membrane and the structure of the cyclic peptide moiety determine the efficacy of iturin (Maget-Dana and Peypoux 1994).

### Bacitracin

**Figure. 7.**
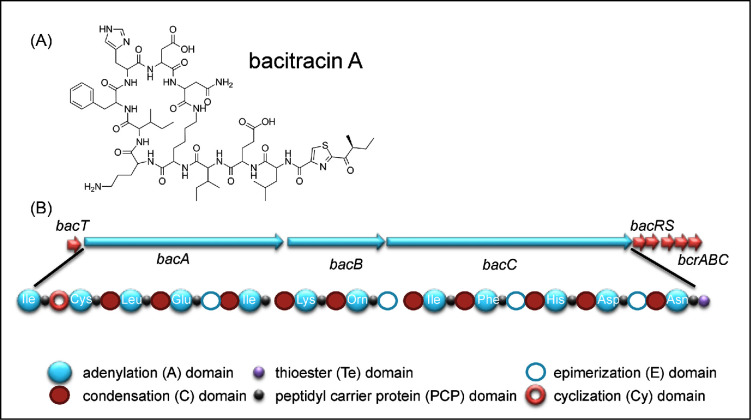
Bacitracin and the bacitracin biosynthesis gene cluster. (**A)** Structure of bacitracin A. (**B**) The bacitracin biosynthesis gene cluster (*bacT,A,B,C,R,S*) from *B. licheniformis* ATCC 10716. BacT, the thioesterase; BacA, BacB and BacC, peptide synthetases; BacR and BacS, two-component regulatory system; BcrA,B,C bacitracin transporter.

Bacitracin is a bacteriostatic, branched cyclic dodecylpeptide metalloantibiotic, bacitracin (Fig. 7) whose synthesis is widely reported to be associated with strains of *B. licheniformis* strains. However, as reported above, analysis of the completed genomes of 14 strains of *B. licheniformis* and 6 strains of *B. paralicheniformis* showed that the bacitracin gene clusters were exclusively associated with the latter (Table 1 and Table S2, Supporting Information). Originally discovered in 1945 (Johnson, Anker and Meleney 1946), bacitracin actually refers to a group of closely related compounds that differ by one or two amino acids. The best studied is bacitracin A, which is the most active against Gram-positive bacteria. Bacitracins inhibit bacterial growth by preventing the dephosphorylation of C_55_-undecaprenyl pyrophosphate (bactoprenol) and thereby the subsequent recycling of the lipid carrier that is essential for cell wall synthesis (Siewert and Strominger 1967).

Synthesis of the core dodecylpeptide is by non-ribosomal synthases in which activated substrate amino acids (amino acyl adenylates) are linked to a 4’-phosphopantetheinyl-cofactor (Konz *et al.*^1997^). Bacitracins contain four amino acids in the D-configuration (Glu4, Orn7, Phe9, Asp11), including the non-proteinogenic amino acid D-ornithine. Cyclisation is the result of condensation of the ε-amino group in lysine and the α-carboxy group of asparagine to form lariat structures (Fig. 7). The 50 kb *B. licheniformis* bacitracin (*bac*) operon includes three genes, *bacA, bacB* and *bacC*, that encode the modular peptide synthetases, *bacT* that encode a TE-like protein and *bacRS* encoding a two-component regulatory system. The bacitracin transporter is encoded by the *bcrABC* operon.

Bacitracin has a low level of toxicity. When administered orally to mice, LD_50_ values are greater than 3750 mg/kg body weight (bw), while administration of 50 mg/kg bw bacitracin by gavage to pregnant rats (7 and 17 days of pregnancy) had no adverse effects on foetal development (EMEA 1998). When applied topically, rashes and anaphylaxis reactions have been recorded in some patients. However, if injected intramuscularly, it can result in tubular and glomerular necrosis and ultimately renal failure. Because infants are much less prone to renal toxicity, this antibiotic is occasionally used in infants to treat pneumonia and empyema. Bacitracin is approved for use in veterinary medicine as a food additive, and in combination with other antibiotics for the treatment of mastitis (Drapeau *et al.*^1992^).

### Bacillibactin

**Figure 8.**
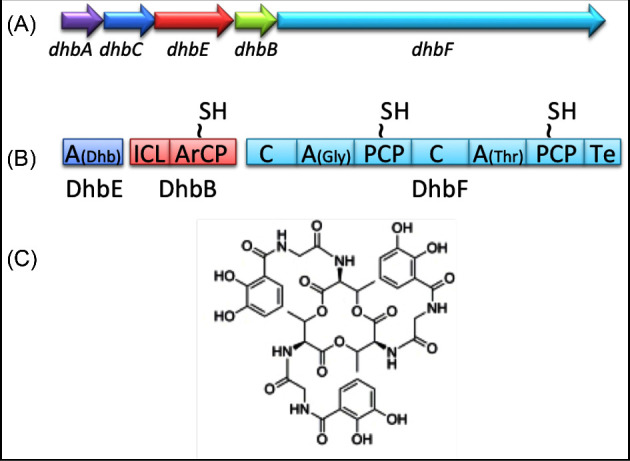
Siderophore biosynthesis gene clusters of *B. subtilis* (*dhb*). (**A**) The *dhbA,C,E,B,F* gene cluster. (**B**) NRP synthetase modules. (**C**) Bacillibactin stucture. A, adenylation domain; ArC and ArcP, aryl carrier protein domain; C, condensation domain; Dhb, 2,3-dihydroxybenzoic acid; Gly, glycine; ICL, isocitrate lyase; PCP, peptidyl carrier protein domain; Te, thioester domain; Thr, threonine (modified from May, Wendrich and Marahiel 2001).

Many members of the genus *Bacillus* synthesise and secrete bacillibactin, a catechol-based hexadentate triscatecholamide siderophore (Fig. 8). The role of this and other siderophores is to obtain iron (Fe^3+^) from the environment and deliver it to the cytoplasm (Hider and Kong 2010). To perform this activity, siderophores have extremely high affinities for iron—in the region of K_f_  =  10^45^ M^−1^. Iron is both an essential nutrient, required for the activity of enzymes involved in important metabolic processes (e.g. respiration DNA synthesis oxidative stress protection), and a highly toxic compound which, if not appropriately stored, generates reactive hydroxyl radicals (^•^OH). Fe^3+^ has low solubility and is therefore difficult to recover from natural environments, while iron withholding is an important element of the innate immune system in which glycoproteins such as transferrin and lactoferrin trap iron to prevent both its acquisition by pathogens and ferrotoxicity (Cassat and Skaar 2013).

Bacillibactin is synthesised via a NRPS complex. Because catecholate siderophores are 2,3-dihydroxybenzoate-containing species, the operon encoding the biosynthetic pathway is named *dhbACEBF* (Fig. 8**A**). The bacillibactin biosynthesis gene clusters of *B. licheniformis* and *B. subtilis* have similar structural organisations and their products show 67%–75% identity. DhbB, DhbE and DhbF are the three modules of the bacillibactin NRPS and their specific enzymatic activities are shown in Fig. 8B (May, Wendrich and Marahiel 2001). DbhC (isochorismate synthase) and DbhA (2,3-dihydro-2,3-dihydroxy benzoate dehydrogenase) are responsible for the synthesis of the 2,3-dihydroxy benzoic acid substrate from chorismate. Bacillibactin is exported via YmfD and Mta (Miethke *et al.*^2006^; Miethke, Schmidt and Marahiel 2008), taken up when Fe^3+^-loaded by the FeuABC transporter, and cleaved intracellularly by the BesA esterase to release its iron.

There are no direct reports of toxicity associated with bacillibactin although this siderophore is an important virulence factor in the case of *B. cereus* and *B. anthracis* where its ability to sequester iron in the presence of host-deployed iron scavenging proteins such as transferrin and lactoferrin is essential for the progression of the infection. In contrast, free iron is potentially toxic, and iron homeostasis is a key element in avoiding ferrotoxicity. Human diseases such as β-thalassemia and sickle cell anaemia can lead to iron overload, and these can be treated with the help of siderophore-based drugs (Chu *et al.*^2010^). Siderophores can also be used as ‘Trojan horses’ to deliver drugs into cells using the siderophore transporter systems (Saha *et al.*^2015^).

## RIBOSOMALLY SYNTHESIZED AND POST-TRANSLATIONALLY MODIFIED PEPTIDES

Relevant examples of ribosomally synthesised and post-translationally modified peptides (RiPPs), also known as ribosomal natural products (RNPs), are briefly discussed because some have structural and functional similarities to PKs and NRPs. RiPPs include bacterially synthesised AMPs that are nearly all cationic and often amphiphilic, reflecting the fact that their antimicrobial activity is often associated with membrane permeabilisation.

### Lantipeptides

Lantipeptides are a group of highly diverse post-translationally modified polycyclic peptides that characteristically contain thioether crosslinks formed by the nonproteinogenic amino acid, lanthionine (Kerr and van der Donk 2012). They are divided into four main classes according to the enzymes responsible of ring formation. Lantipeptides are synthesised as precursor peptides comprising a leader peptide and core peptide. The precursor is postranslationally modified, thioether cross-linked and then the leader peptide removed prior to release of the mature lantipeptide.

Many lantipeptides have antimicrobial activity and indeed the prototypical lantipeptide, nisin, is widely used commercially as a food preservative. The antimicrobial activity of lantipeptides, where known, is often associated with the disruption of cell membrane integrity or cell wall biosynthesis. For example, nisin targets and sequesters lipid II, both blocking peptidoglycan transglycosylation and the formation of stable membrane-spanning pores (Wiedemann *et al.*^2001^).

A number of gene clusters involved in lantipeptide synthesis and maturity were identified in the complete genomes of *B. amyloliquefaciens, B. licheniformis* and *B. subtilis* using antiSMASH*;* however, their specific products are often not specifically identified. More recently, Walsh *et al.* (2017) have developed a search tool, based on a Profile hidden Markov model (profile-HMM), for the improved identification of class 1 lantibiotic gene clusters in metagenomic data.

**Figure 9.**
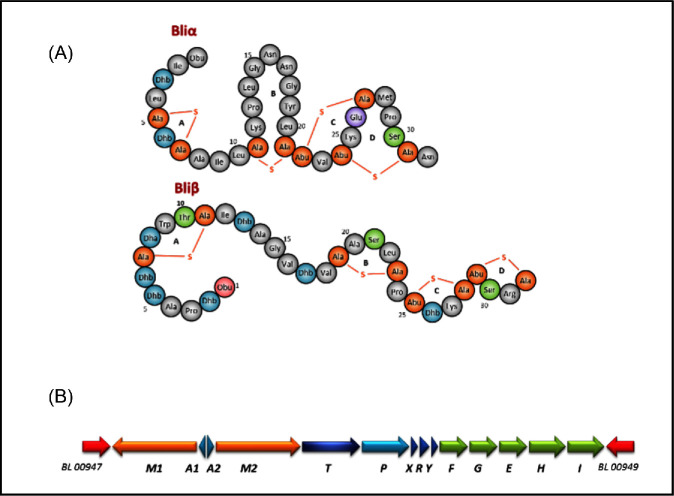
Lichenicidin VK21 and the lichenicidin gene cluster. (**A**) Structure of lichenicidin VK21. (**B**) Lichenicidin gene cluster responsible for synthesis, export and immunity (modified from http://www.biochemie.tu-berlin.de/Research/ResearchClassIILanthipeptides2/Research-ClassIILanthipeptides2.xhtml).

*Bacillus licheniformis* strains have a gene cluster encoding the lantipeptide, lichenicidin, a two-peptide lantibiotic (Fig. 9A) that targets lipid II (Begley *et al.*^2009^; Dischinger *et al.*^2009^; Shenkarev *et al.*^2010^). Lichenicidin is synthesised as two propeptides that are matured by the removal of sequences at their N-termini. Subsequent post-translational modifications introduce lanthionine and methyllanthionine and, in the case of lichenicidin VK21, each of the 32-amino acid long peptides is cross-linked by four intramolecular thioether bridges and has an N-terminal 2-oxobutyryl group (Fig. 9A). Together, the mature peptides, Bliα and Bliβ, are active against Gram-positive bacteria in the nanomolar concentration range, while individually the peptides are active at the micromolar concentration range.

The *B. licheniformis* genes encoding the lichenicidin propeptides are annotated as either *lin* (lichenicidin) or *lan* (lantibiotic). Genes *lic/lanA1* and *lic/lanA2* encode the propeptides LanA1 (42 amino acids) and LanA2 (34 amino acids) with Gly-Gly-like cleavage site motifs (Fig. 9B). The products of *LicM1* and *LicM2* are modification enzymes responsible for the formation of the lanthionine residues: Ser/Cys residues are modified to lanthionine, while Thr/Cys residues are modified to methyllanthionine. The product of *licT* is a membrane-spanning transporter with an N-terminal protease domain required to cleave the leader peptides of the precursors during translocation across the membrane. After transport, the product of *licP*, an extracellular protease, removes six residues from Bliβ. Finally, the *licFGEHI* operon encodes the proteins necessary for self-immunity.

### Sublancin 168

**Figure 10.**
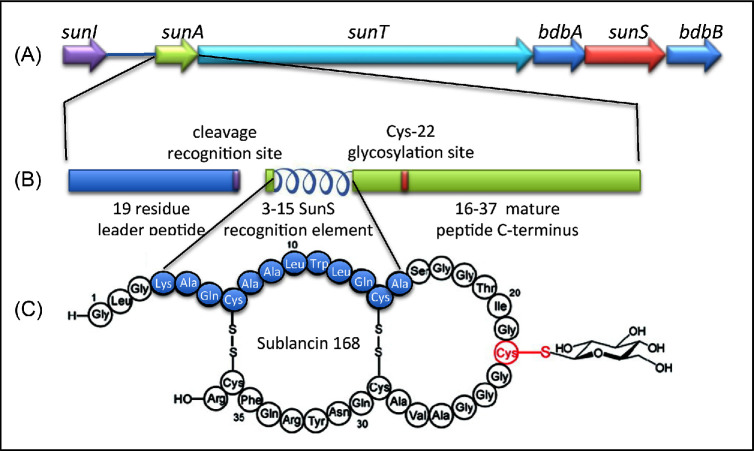
The sublancin 168 operon and glycopeptides structure. (**A**) *Bacillus subtilis* sublancin operon encoding SunI, immunity protein; SunA, propeptide; SunT, sublancin transporter, BdbAB, disulphide bond proteins; SunS, S-glycosyltransferase. (**B**) Sublancin 168 propeptide with leader peptide and G-G cleavage site, and mature peptide with SunS recognition site and Cyc-22 glycosylation site. (**C**) Structure of sublancin 168 (modified from Hsieh *et al.*^2012^).

*Bacillus subtilis* 168 encodes sublancin 168, a 37-residue glycosylated peptide. The sublancin (*sun*) operon (*sunAT, bdbA, sunS, bdbB*) is located on the genome within the SPβ prophage. Although widely reported to be a lantibiotic (Fig. 10), more recent work has shown it to be a member of a small group of S-linked glycosylated AMPs known as glycocins (Oman *et al.*^2011^). This group of AMPs is unusual in having a glucose moiety β-linked to cysteine at position 22. Sublancin is synthesised as a precursor peptide, SunA. In addition to *sunA*, the *sun* operon encodes the S-glycosyltransferase (SunS), responsible for glycosylation, an ABC transporter (SunT) that is responsible for its export and removal of its signal peptide, and two thiol-disulfide oxidoreductases, BdbA and BdbB, the latter being required for disulphide bridge formation (Dorenbos *et al.*^2002^; Hsieh *et al.*^2012^). Upstream of the sublancin operon is a monogenic operon encoding the SunI immunity protein.

Sublancin 168 is active against Gram-positive bacteria, albeit with varying degrees of sensitivity. It targets the phosphoenolpyruvate:sugar phosphotransferase system, with the cognate substrate sugar decreasing sensitivity (De Gonzalo *et al.*^2015^). The sublancin structure has two α-helices and a nine-residue interhelical loop (Fig. 10C) with a glucose moiety attached at position Cys22. Its three-dimensional structure provides sublancin 168 with an extraordinary high degree of stability (De Gonzalo *et al.*^2014^).

### Lassopeptides

Lassopeptide gene clusters are often identified in the genome of members of the *B. subtilis* group, although their products are often not identified specifically. However, Tietz *et al.* (2017) used RODEO, a new HMM-based genome-mining tool for identifying RiPPs, to redefine the lassopeptide biosynthetic landscape. Lassopeptides are potent AMPs that are characterised by the presence of a macrolactam ring at the N-terminus that traps a C-terminal tail (Weber *et al.*^1991^). Lassopeptide biosynthesis involves the synthesis of the precursor A-peptide that is post-translationally modified by the B-protein, an ATP-dependent cysteine protease that removes the leader peptide, and the C-protein, an ATP-dependent asparagine synthetase that catalyses the formation of a macrolactam ring between the N-terminal amino group and the side chain of an aspartate or glutamate residue in the peptide. The latter reaction is catalysed in such a way as to localise the C-terminal peptide tail within the ring, giving rise to the name giving ‘lasso structure’. This structure is further stabilised by the presence of bulky plug residues within the peptide tail and disulfide bonds.

## SAFETY AND TOXICITY

The use of microorganisms for the production of food and feed additives (e.g. enzymes vitamins pre- or probiotics, etc.) has been of major benefit to agriculture, farming and primary food production. However, microbes producing secondary metabolites need to be regulated to ensure that they are both safe and do not add to the burden of resistance to antibiotics use in the clinic. In the European Union, Regulation (EC) N^o^ 1831/2003 establishes the rules governing the authorisation of feed additives for use in animal nutrition, and this is enacted through the European Food Standards Authority's (EFSA) panel on Additives and Products or Substances used in Animal Feed (FEEDAP; EFSA FEEDAP 2011).

In response to the need of EFSA to establish a generic approach for assessing the safety of microorganisms and additives used in food and feed, its Scientific Committee publishes a list of microorganisms recommended for Qualified Presumption of Safety (QPS) (EFSA 2005; 2007). The list initially consisted of 48 species of non-sporulating Gram-positive bacteria, 13 species of spore-forming Gram-positive bacteria and 11 species of yeast. This list has been regularly updated and the 2017 update comprises 58 species of non-sporulating Gram-positive bacteria, 15 species of spore-forming Gram-positive bacteria (*Bacillus* and *Geobacillus* species), 2 species of Gram-negative bacteria (*Gluconobacter oxydans* and *Xanthomonas campestris*), 14 species of yeast and 3 virus families (EFSA BIOHAZ 2017). Both *Bacillus licheniformis* and *B. subtilis* have QPS status with the qualifications that they do not harbour any acquired antimicrobial resistance genes to clinically relevant antimicrobials or exhibit toxigenic activity.

The safety of *Bacillus* species has been extensively reviewed (de Boer and Diderichsen 1991; Ishibashi and Yamazaki 2001; Sanders, Morelli and Tompkins 2003). A few cases of toxicity concerning members of the *B. subtilis* group have been reported, although the evidence for their involvement tends to be circumstantial rather than unambiguously proven. In some cases, the provenance of the strains involved has been questioned (de Boer and Diderichsen 1991; Drobniewshi 1993; Agerholm, Krogh and Jensen 1995; Salkinoja-Salonen *et al.*^1999^; From *et al.*^2007^). There are also reports of opportunistic *B. subtilis* infections in immuno-compromised patients (Oggioni, Pozzi and Valensin 1998).

It is not surprising that reports associating QPS strains with disease are rare since virulence is rarely, if ever, a monofactorial phenomenon. Instead pathogens elaborate a series of virulence factors that aid access to target sites within the host, help resist or evade the immune systems and generally promote survival in the host. For example, in the case of *B. cereus sensu stricto*, a key component of virulence is PlcR, a regulator of the large number of virulence factors encoded by this bacterium. PlcR regulon members include genes encoding enterotoxins NheA, NheB, NheC, HblB, HblL1 and HblL2, haemolysins Clo and CytK, phospholipases PlcA, PlcB and Smase, neutral proteases NprP2, NprC and NprB, collagenases ColA and ColC, metalloprotease InhA2, proteases MpbE and Sfp, AMPs, SppC1 Sppc2 and SppC3, and a drug efflux protein (Gohar *et al.*^2008^). In contrast, no homologue of PlcR has been identified in any member of the *B. subtilis* group and nor is there evidence of well-characterised virulence factors. Such analyses have been considerably helped by the increased speed, and reduction in cost, of genome sequencing.

Article 4(1) of EC regulation 1831/20032 requires enterprises to seek authorisation for the use of additives to food or feed. Although widely used commercial strains of *B.* subtilis and *B. licheniformis* produce well-characterised secondary metabolites (PKs and NRPs) and AMPs, there are no well-authenticated reports of human or animal toxicity associated with these compounds. Indeed each year the Japanese consume ∼7 billion helpings of natto, a soybean-based food fermented using a surfactin-producing natto variant of *B. subtilis.* Nevertheless, three generic issues that are of particular relevance to regulatory authorities and industry, and that have led to previous ambiguities in the literature, are as follows: (i) the historical mis-identification of strains, (ii) the terminology used to describe these compounds and (iii) the assays used to determine their toxicity.

Prior to authorisation for use as a food/feed additive, strains belonging to the *B. subtilis* group must be tested for the production of toxins similar to the haemolytic (HBL) and non-haemolytic (NHE) enterotoxins of *B. cereus*, as well as the emetic toxin cereulide (Pedersen *et al.*^2002^). While toxigenic assays and PCR-based diagnostic tools have demonstrable merit, their data must be interpreted with care (see below). Similarly, while the presence of genes encoding these and other well established toxins can be identified from whole genome sequence data, this too is not without its limitations due to the absence of quality control metrics (Ellington *et al.*^2017^). For example, there is a single report of the detection of the *B. cereus* haemolytic (*hblACD)* and non-haemolytic (*nheABC*) genes in *B. licheniformis* strain N662. However, analysis of the amplified gene fragments showed that they were 99% identical those in members of the *B. cereus* group (de Bellis *et al.*^2015^). When we used BLASTn to search for homologues of these genes among all *B. licheniformis* (taxid: 1402) sequences in the NCBI database, the only sequences that were identified were from strain N662. In contrast, when no taxonomic limitations were applied to the BLASTn analysis, aside from strain N662, all other strains showing homology were identified as being members of the *B. cereus sensu lato* group. In the absence of a whole genome sequence data, this suggests that (i) strain N662 has acquired the *hblACD* and *nheABC* genes recently as the result of two independent horizontal gene transfer events, or (ii) the strain itself has been misidentified or (iii) the chromosomal DNA used in the PCR was contaminated with DNA from other strains used in the study.

It is questionable whether assays devised to screen extracts from strains previously known to produce well-characterised cytotoxins (e.g. the NRP cereulide from *B. cereus*) are necessarily appropriate for distinguishing between cytotoxins and cytolysins. Assays that have been used to identify toxicity include haemolysis, the loss of boar spermatozoan motility and inhibition of Vero cells and Chinese Hamster Ovary (CHO) cells (Sandvig and Olsnes 1982; Andersson *et al.*^1998^; Beattie and Williams 1999). The precise molecular mechanisms underlying these bioassays are often unclear and consequently so too are their specificities. For example, inhibition of CHO cells, as determined with the redox dye MTT (3-(4,5-dimethylthiazol-2-yl)-2,5-diphenyl tetrazolium bromide), was successful not only in detecting the cytotoxigenic activity of known enterotoxigenic and emetic strains of *B. cereus* (Beattie and Williams 1999), but also gave positive results for representative strains of *B. licheniformis* and *B. subtilis.* This observation was not followed up; however, the most likely explanation is the failure of this assay to distinguish between cytotoxicity and cytolysis. Consequently, while the CHO-MTT and similar assays (e.g. boar spermatozoa motility and Vero cell assays) are useful for detecting the presence of known cytotoxins, as in the case of cereulide (Pedersen *et al.*^2002^; Toh *et al.*^2004^), their lack of specificity means that it is not valid to extrapolate ‘positive’ results to other organisms. For example, a positive result could be obtained for any surface-active compound that permeabilises cell membranes, such as anionic surfactants (e.g. lichenysin and surfactin in the case of *B. licheniformis* and *B. subtilis*) and detergents (e.g. SLS). Ultimately this points to the need to distinguish clearly between specific cytotoxicity and more general cytolysis.

Strains that produce antimicrobial compounds (e.g. NRPs, PKs and RiPPs) should have their inhibitory compounds identified to determine whether they are relevant to use in humans and animals, and the WHO regularly updates the list of antimicrobials that are of critical important for human medicine (WHO AGISAR 2017). For production strains in which antimicrobial activity has been identified, it is important to demonstrate the absence of carry over into the final product. Of relevance to *Bacillus* species are lipopeptides and polymyxins. Lipopeptides are categorised as prioritisation criterion 1 (P1) on the basis of their widespread use, while polymyxins fall into categories P1 to P3 depending on type. The most significant of the polymyxins is colistin, which is categorised as P3. Colistin is the fifth most popular antibiotic used on production animals in Europe (Rhouma, Beaudry and Letellier 2016) and is also a last-resort antibiotic used for the treatment of severe bacterial infections in humans in cases where other antibiotics have lost their efficacy due to resistance. With the discovery of colistin resistance, mediated by the *mcr-1* gene, there is concern that resistance that may have developed in production animals could be transmitted to humans via the food chain. Colistin is produced naturally by *Paenibacillus polymyxa* but not by either *B. licheniformis* or *B. subtilis.*

Finally in response to an EFSA call to tender, a database on the taxonomical identification and potential toxigenic capacities has been developed for production strains without QPS (de Benito *et al.*^2017^). As a result, members of the *B. subtilis* group are specifically not included. However, the report associated with the database is of generic value by addressing five key objectives in relation to potential production strains and their products:

1. A description of the current valid scientific names, taxon assignation, synonyms and methods for the taxonomic description of microorganisms.
2. The identification of toxins or potentially toxic secondary metabolites/substances produced by the microorganisms used to produce food enzymes and feed additives.
3. Identification of the conditions under which the toxic compounds are produced.
4. The characterisation of toxic compounds.
5. The biosynthetic pathways and genetic characterisation of the toxic compounds.

The review by de Benito et al. (2017) also includes a valuable list of keywords for searching the literature for terms relating to toxin production and hazards; feed additives and food enzymes; fermentation processes; toxicity; biosynthetic pathways.

## CONCLUDING REMARKS

Industrial microorganisms such as *B. subtilis* and *B. licheniformis* produce a range of secondary metabolites and AMPs that improve survival in their native environments. These metabolites are synthesised via a variety of pathways that are not only responsible for the synthesis of the core elements but also facilitate extensive modifications that vastly increase the range of molecular structures that are observed in nature. This represents a challenge because currently, while bioinformatics can predict with reasonable accuracy the core structures, precise structural identification still requires detailed chemical analyses.

Many of these secondary metabolites are of value as antibacterial and antifungal compounds and, as a result, certain *B. subtilis* group strains have been developed as probiotics and for the production of AMPs and other metabolites for use in humans and animals (Cutting 2010). A complicating factor is the production, by species related to the *B. subtilis* group, of toxigenic cyclic peptides, such as the emetic toxin cereulide produced by strains of *B. cereus.* While the action of this toxin is highly specific, the assays used to detect its presence are not, and their use to determine the toxicity of other secondary metabolites and AMPs has led to inconsistencies in the literature and the need to review criteria concerning toxicity (EFSA FEEDAP 2014).

Companies producing strains and products authorised as additives in food and feed have a vested interest in ensuring the safety of their products. Strains used for industrial purposes have been developed and used for decades and therefore have a comprehensive history of safe usage, backed by numerous toxicity studies. As part of their quality assurance policy, enzyme manufacturers routinely analyse their strains and products for the presence of compounds that might compromise their safety. As a result, there is a considerable body of evidence, often accumulated over decades, relating to the safe use of *B. licheniformis*, *B. subtilis* and related strains for enzyme production and as food/feed additives.

## Supplementary Material

Supplement Tables
